# Association between Chronic Gingivitis and Cancer: A Retrospective Cohort Study of 19,782 Outpatients from the United Kingdom

**DOI:** 10.3390/cancers15072007

**Published:** 2023-03-28

**Authors:** Jane Beger-Luedde, Sven H. Loosen, Tom Luedde, Christoph Roderburg, Karel Kostev

**Affiliations:** 1 Practice Centre for Orthodontics, Laurensberg, 52072 Aachen, Germany; jane.beger@gmx.de; 2 Clinic for Gastroenterology, Hepatology and Infectious Diseases, University Hospital Düsseldorf, Medical Faculty of Heinrich Heine University Düsseldorf, 40225 Düsseldorf, Germany; sven.loosen@med.uni-duesseldorf.de (S.H.L.); tom.luedde@med.uni-duesseldorf.de (T.L.); 3 Epidemiology, IQVIA, 60549 Frankfurt, Germany; karel.kostev@iqvia.com

**Keywords:** gingivitis, cancer, biomarker, prostate cancer, age

## Abstract

**Simple Summary:**

Gingivitis was recently suggested as a new risk factor for systemic inflammation. Here, we show that chronic gingivitis represents an important risk factor for the development of cancer. These data suggest that gingivitis patients should be included in existing screening programs for early detection and prevention of cancer.

**Abstract:**

**Purpose:** Recent data argue for the involvement of inflammatory and infectious diseases in cancer development. However, clinical data on the association between chronic gingivitis and cancer have been less conclusive. Here, we systematically evaluated the cancer incidence in a population-based cohort of outpatients with chronic gingivitis from the United Kingdom. **Methods:** 9891 patients with chronic gingivitis and an identical number of people without gingivitis matched by age, gender, index year, and the Charlson Comorbidity Index were identified from the Disease Analyzer database (IQVIA) between January 2000 and December 2016. Cox regression models were used to study the association between gingivitis and cancer. **Results:** The probability of cancer was significantly higher among patients with diagnosed chronic gingivitis compared to non-gingivitis individuals (HR = 1.36, 95% CI = 1.15–1.62). In cancer site-stratified analyses, we observed a trend towards higher rates of cancer in almost all cancers (breast cancer, lymphoid system cancer, digestive tract cancers, skin cancer); however, a significant association was only observed for prostate cancer (HR: 3.38; 95% CI: 1.57–7.27). Notably, the largest increase in cancer rates was observed in male patients (HR: 1.46, 95% CI: 1.13–1.89) between 41 and 60 years old (HR: 1.74, 95% CI: 1.30–2.32). **Conclusions:** Our data suggest that chronic gingivitis represents an important risk factor for the development of cancer. Therefore, in the context of patient dental care, awareness should be raised to refer gingivitis patients to existing screening programs, especially for prostate cancer. Moreover, the consistent treatment of gingivitis could potentially have a positive impact on the morbidity of certain cancers.

## 1. Introduction

Periodontal diseases include a number of diseases such as gingivitis and periodontitis that are associated with damage to the gums and periodontium. Periodontal diseases represent a very common clinical issue worldwide [1]. Most authors suggest that periodontal disease is caused by the accumulation of plaque, a sticky film of bacteria, on the teeth and gums. This plaque causes inflammation of the gums, eventually leading to gingivitis. If left untreated, plaque can spread below the gum line and lead to periodontitis, causing the gums to pull away from the teeth, forming pockets. Bacteria can grow in these pockets, causing bone loss and eventually leading to tooth loss. Risk factors for periodontal disease include tobacco use, poor oral hygiene, certain medications, genetics, and underlying medical conditions such as diabetes [1]. In the United States, the prevalence of periodontitis in adults over the age of 30 years is estimated to be around 47% and increases to 70% in individuals 65 years of age or older [1,2], with similar numbers reported from European countries [3].

Along with local complications, including bleeding and tooth loss, periodontal diseases have been linked to several systemic diseases and conditions, making them a significant risk factor [4], which is not sufficiently recognized by most patients [5,6]. Studies have shown that periodontal diseases can increase the risk of cardiovascular diseases, ankylosing spondylitis, diabetes, respiratory diseases, and chronic kidney disease [7,8,9,10,11,12]. The presence of periodontal disease has also been associated with adverse pregnancy outcomes, such as preterm birth and low birth weight [13,14]. Additionally, research suggests that periodontal disease may play a role in the development and progression of Alzheimer’s disease and other forms of dementia. Therefore, periodontal diseases can have a significant impact on overall health and well-being, and it is important for individuals to maintain good oral hygiene and seek timely treatment for periodontal problems to reduce the risk of these and other associated diseases.

Nevertheless, questions remain regarding the strength and magnitude of this association as well as the entity-specific risk for cancer development, because available studies were hampered by potential methodological limitations, including the use of self-reported diagnoses of periodontal disease [15] or small sample sizes [16]. In light of this lack of comprehensive data, the aim of this study was to provide epidemiologic evidence for the association between gingivitis and cancer risk. We, therefore, used the Disease Analyzer database (IQVIA), featuring drug prescriptions, diagnoses, and basic medical and demographic data from general practitioners in the United Kingdom (UK) of about 4,000,000 patients, to dissect the association between chronic gingivitis and the most frequent types of cancers. We demonstrate that chronic gingivitis represents a major risk factor for the development of cancer in humans. A particularly strong association was found for prostate carcinoma, highlighting a novel group with a strong need for cancer prevention and early detection programs.

## 2. Methods

### 2.1. Database

This study was based on data from the Disease Analyzer database UK (IQVIA), which has been described in detail elsewhere [17]. The database contains anonymized electronic patient records obtained directly and in anonymized format from computer systems used in the practices of general practitioners in the UK. Patient data were analyzed in aggregated form without individual data being available. All methods were carried out in accordance with relevant guidelines and regulations.

### 2.2. Study Population

This study included patients who had received an initial diagnosis of chronic gingivitis (ICD-10: K05.1) in one of 256 general practices (1721 physicians) in the United Kingdom between January 2000 and December 2016 (index date). Inclusion criteria were as follows: patient age of ≥18 years at the index date and no cancer (ICD-10: C00–C99), in situ neoplasms (ICD-10: D00–D09), and neoplasms of uncertain or unknown behavior (ICD-10: D37–D48) diagnoses documented prior to the index date.

**Figure 1:**
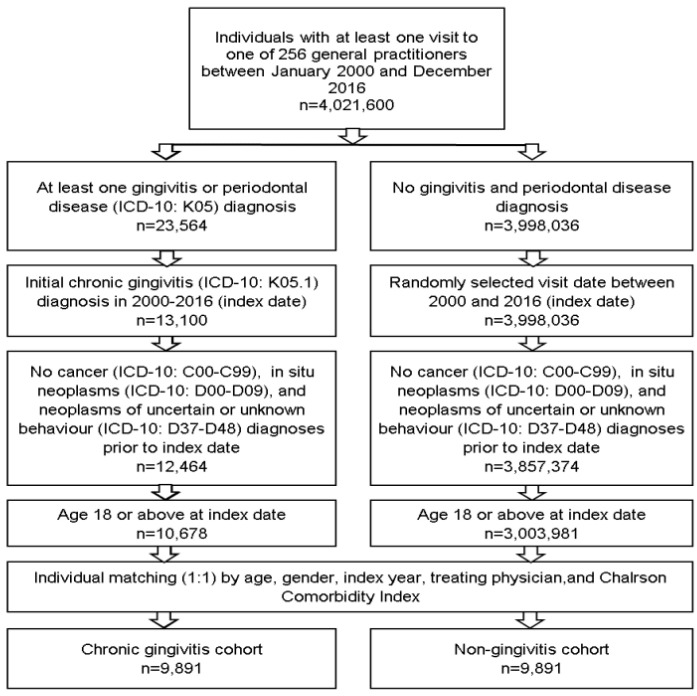
Selection of study patients.

After applying similar inclusion criteria, patients without chronic gingivitis were matched (1:1) to those with chronic gingivitis by sex, age, index year, treating physician, and the Charlson Comorbidity Index. The Charlson comorbidity index describes 22 comorbid conditions, where each condition is assigned a score from 1 to 6 depending on the risk of mortality. For patients without chronic gingivitis, the index date was a randomly selected visit date between January 2000 and December 2016 (Figure 1).

### 2.3. Study Outcome and Statistical Analyses

We analyzed the cumulative incidence of cancer in patients with chronic gingivitis compared to patients without chronic gingivitis as the primary outcome. Baseline characteristics were compared between patients with and without chronic gingivitis using McNemar tests for categorical variables and Wilcoxon signed rank tests for continuous variables. The association between chronic gingivitis and cancer was studied with Cox regression. Different types of cancer ICD 10: C00–C14 (malignant neoplasms of lip, oral cavity, and pharynx); C15–C26 (malignant neoplasms of digestive organs); C30–C39 (malignant neoplasms of respiratory and intrathoracic organs); C43 (malignant melanoma), C44 (other malignant neoplasms of skin); C50 (malignant neoplasm of the breast); C51–C58 (malignant neoplasms of female genital organs); C61 (malignant neoplasm of the prostate); C64–C68 (malignant neoplasms of the urinary tract); C81–C96 (malignant neoplasms of lymphoid, hematopoietic and related tissue) were separately analyzed as a function of gingivitis. Analyses were performed using SAS version 9.4 (SAS Institute, Cary, NC, USA).

## 3. Results

### 3.1. Basic Characteristics of the Study Sample

In this study, 9891 patients with chronic gingivitis were matched to 9891 patients without gingivitis (1:1 matching). The proportion of women in the population was 56.9%, and the mean (standard deviation) age was 41.6 (17.6) years (Table 1). Most patients belonged to the age group between 18 and 40 years (53.8%). Furthermore, 28.9% of the patients were between 41 and 60 years old, and 17.3% of the patients were older than 60 years. The age distribution was identical between the group of patients with gingivitis who developed a tumor during the observation period and the group who did not develop a tumor. Furthermore, the Charlson comorbidity index, as a surrogate marker for the general patients’ condition and the degree of concomitant patients´ diseases, was equally distributed between both groups.

### 3.2. Gingivitis Is Associated with an Increased Incidence of Cancer

**Figure 2:**
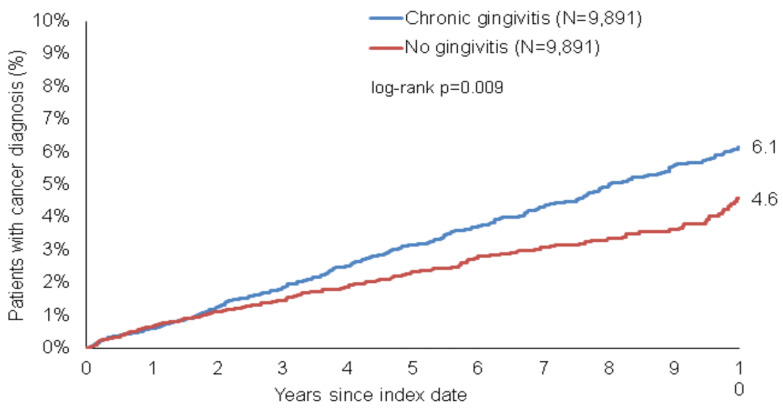
Cumulative incidence of cancer in patients with and without chronic gingivitis in the 10 years following the index date.

We first aimed at evaluating the association between chronic gingivitis and the development of cancer in general. During the ten-year observation period, 6.1% of individuals with gingivitis and 4.1% of patients without gingivitis were diagnosed with cancer (log-rank, *p* = 0.009, Figure 2). Already early in the observation period, an increased incidence of malignancy in patients with gingivitis became apparent; notably, this difference further increased during the observation period. Cox regression analysis confirmed the positive and significant association between gingivitis and cancer (HR: 1.36, 95% CI = 1.15–1.62, *p* < 0.001, Table 2).

### 3.3. Age- and Sex Stratified Analyses

Based on these highly promising data, which highlight an association between chronic gingivitis and the development of all-site cancer, in a next step, we aimed at evaluating potential age- or sex-related associations between gingivitis and cancer. In this analysis, the positive association between both diseases was stronger in men (HR: 1.46, 95% CI: 1.13–1.89, *p* = 0.004) compared to female patients (HR: 1.29, 95% CI: 1.03–1.62, *p* = 0.029, Table 2). In age-stratified analyses, the positive association between gingivitis and cancer was strongest in patients aged between 41 and 60 years (HR: 1.74, 95% CI: 1.30–2.32, *p* < 0.001, Table 2). Notably, a trend towards higher cancer rates was observed in all analyzed subgroups, supporting the role of gingivitis as an age- and sex-independent risk factor for cancer development in humans.

### 3.4. Cancer Site Stratified Analyses

**Figure 3:**
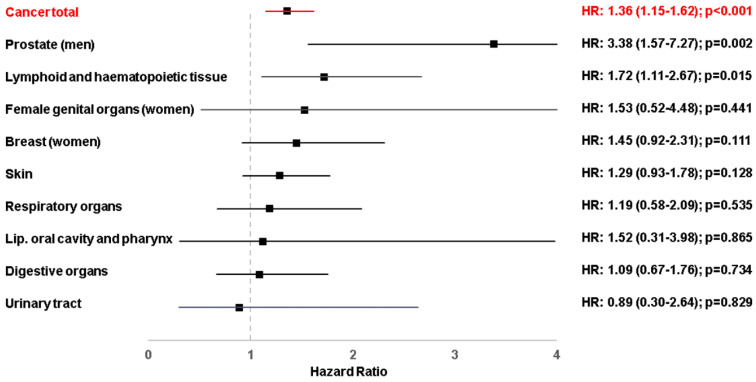
Association between chronic gingivitis and the incidence of different cancers in patients followed for up to 10 years in general practices in the United Kingdom.

Finally, we aimed to further dissect a potential association between gingivitis and various defined cancer sites. Although the comparatively small sample sizes did not allow to identify significant associations in most cancer-site stratified analyses, we observed a trend toward a positive relationship between gingivitis and almost all distinct cancer types. However, a strong and statistically significant association was only observed for prostate cancer (HR: 3.38; 95% CI: 1.57–7.27, *p* = 0.002). Moreover, for lymphoid and hematopoietic malignancies, a strong and nearly significant association was observed (HR: 1.72; 95% CI: 1.11–2.67, *p* = 0.015; Figure 3). In contrast, we did not observe a significant association between gingivitis and female genital organ cancer (HR: 1.53; 95% CI: 0.52–4.48, *p* = 0.441), breast cancer (HR: 1.45; 95% CI: 0.92–2.31, *p* = 0.111), skin cancer (HR: 1.29; 95% CI: 0.93–1.78, *p* = 0.128), respiratory organ cancer (HR: 1.19; 95% CI: 0.58–2.09, *p* = 0.535), cancer of the lip, oral cavity, and pharynx (HR: 1.52; 95% CI: 0.31–3.98, *p* = 0.865) or digestive organ cancer (HR: 1.09; 95% CI: 0.67–1.76, *p* = 0.734, Figure 3). Altogether, these data clearly suggest a link between the presence of chronic gingivitis and the development of cancer that should be discussed with the respective patients in order to provide an optimal and holistic treatment.

## 4. Discussion

Periodontal disease, including the two medical conditions gingivitis and periodontitis, represents a major global health burden with a high prevalence, especially in older patients [1]. Both diseases are characterized by an ongoing inflammation within the oral cavity as well as systemic inflammation status, which can lead to “systemic sequela”, including cardiovascular diseases, diabetes, respiratory diseases, and chronic kidney disease [7,8,9,10,11,12]. Moreover, both experimental and epidemiologic studies previously suggested an increased incidence of cancer in patients with chronic gingivitis, but many questions, including the statistical strength of the associations and the specific profile of malignancies, have remained open.

Here, we demonstrate in a large, well-characterized, and well-matched outpatient population of over 19,000 patients from the UK that the probability of cancer is significantly higher among patients with chronic gingivitis compared to patients without. Interestingly, this effect was most apparent in male patients between 41 and 60 years old and in patients with prostate cancer.

Several human epidemiological studies and experimental animal models suggest an influence of periodontal diseases and chronic gingivitis on the activation of systemic inflammatory processes and immune responses. As such, patients with chronic gingivitis have higher serum concentrations of inflammatory markers such as C-reactive protein [18,19,20]. The link between systemic inflammation and the development of chronic diseases is well established and may contribute to the strong and consistent positive associations between chronic gingivitis and metabolic diseases such as diabetes and cardiovascular diseases [21,22]. Interestingly, metabolic and malignant diseases share many risk factors, including smoking, obesity, lack of physical activity, insulin resistance, and diabetes [23], which have triggered different lines of research to address the role of periodontal disease in promoting the development of malignancies [4].

In this context, our data are in line with previous analyses demonstrating increased cancer rates in patients with periodontal diseases [4,24]. Similar to many of these previous studies, we were unable to control our data for the patients´ smoking status. Nevertheless, our analyses revealed an HR of 1.36 for the development of all-site cancer, corroborating recent data from studies that controlled for smoking and demonstrated that periodontal disease is associated with an increased total cancer risk of 14% to 20% [15,25]. In our cohort of nearly 10,000 patients with chronic gingivitis and a similar number of matched non-gingivitis individuals, the positive and significant association between gingivitis was substantially carried by the greatly increased risk for prostate cancer. This finding is of particular interest due to the fact that prostate cancer is not a smoking-associated cancer and thus supports the hypothesis that systemic effects of chronic gingivitis might drive cancer development. Michaud et al. recently presented data from 7466 participants in the Atherosclerosis Risk in Communities (ARIC) study cohort, showing that cancer risk, especially for lung and colorectal cancer, is elevated in individuals with periodontitis [26]. In addition, a recent meta-analysis comprising 50 studies from 46 publications provided support for a positive association between periodontal disease and the risk of oral, lung, and pancreatic cancers [4]. Thus, our data nicely add to the available literature by establishing prostate cancer as another malignant disease deserving clinical attention in patients with periodontal diseases.

The significant association between chronic gingivitis and prostate cancer raises the question of a pathophysiological basis for this mere descriptive observation. Interestingly, both local inflammation (prostatitis) as well as systemic inflammation have been associated with the pathophysiology of prostate cancer development [27,28]. In this line of thinking, it is of importance that periodontitis has been previously associated with prostatitis [29], which could point towards a link between gingivitis and prostatitis potentially representing an underlying cause for the association between gingivitis and prostate cancer that we observed in our cohort of patients. On a more detailed level, there is evidence that periodontal disease could increase circulating levels of certain pro-inflammatory cytokines, including IL-6, IL-1β, and TNF-α, that were also found to be upregulated in serum samples of men with prostatitis [29]. This could support the hypothesis that periodontitis and/or gingivitis indirectly contributes to the development or substantiation of prostatitis [30,31]. The periodontium has also been described as a distant non-prostatitis source of prostate-specific antigen (PSA) [32], and both a larger study from China [33] as well as an investigation from India [29] have found a relationship between periodontitis and PSA levels in patients with chronic prostatitis. Moreover, another study managed to simultaneously detect the DNA of two oral pathogens, Porphyromonas gingivalis and Treponema denticola, in subgingival plaques as well as in the prostatic fluid of men with periodontal and prostatic disease [34]. Most strikingly, a group from the United States just recently showed that periodontal treatment can improve prostate symptoms and lower serum PSA levels in men with high baseline PSA concentrations and chronic periodontitis [35].

As pointed out, chronic gingivitis is a condition characterized by inflammation of the gums that can last for a prolonged period of time. It is caused by the buildup of plaque on the teeth, which can lead to the formation of bacterial colonies that produce toxins that irritate the gums [36,37]. Chronic inflammation and damage to the cells in the gums were suggested to increase the risk for oral cancer development. Notably, in our analyses, this association was only weak. While the exact mechanism linking gingivitis with oral cancer is not yet fully understood, it is thought that the chronic inflammation and damage to the cells, as well as strains of bacteria associated with chronic gingivitis, such as Porphyromonas gingivalis, may promote the growth of cancer cells or impair the body’s immune response to cancer cells [38]. Notably, it has been found that these bacteria produce enzymes that can damage DNA in the cells of the oral cavity, potentially increasing the risk of genetic mutations that could lead to the development of oral malignancies [38]. Of note, it is important to highlight that other risk factors for oral cancer, such as tobacco and alcohol use, are considered to be much more significant factors in the development of the disease (e.g., [39]).

Our study was limited by some aspects, which are mainly related to the study design and methods and cannot be avoided. Relying on ICD-10 codes might be associated with a misclassification and undercoding of specific diagnoses since it seems likely that rare and highly specific diagnoses will be underrepresented. In this context, it should be noted that many individuals are unaware that they suffer from gingivitis and do not seek medical help despite having the disease. Similarly, the database does not contain any information regarding the technical details of how the diagnosis “gingivitis” was made, so misdiagnoses and false diagnoses cannot be ruled out. However, the IQVIA Disease Analyzer database has proven its statistical validity in numerous previous publications [40,41,42,43,44,45]. Moreover, data on the socioeconomic status (e.g., education and income of patients) as well as lifestyle-related risk factors (e.g., smoking status, physical activity) are also lacking. This is of particular relevance since, e.g., alcohol intake or tobacco abuse represent strong risk factors for the development of any site cancer and gingivitis as well. Similarly, we cannot exclude that some co-morbidities, mainly other kinds of inflammatory diseases, can interfere with and be linked to cancer. Since our study was based on a small sample of gingivitis patients, we cannot exclude the possibility that the documentation of gingivitis was incomplete. Other studies with similar study designs using other databases from other countries also need to be performed before final conclusions can be drawn from our data. Finally, we cannot exclude a selection bias in our study for those with a diagnosis of chronic gingivitis, meaning that patients who have an established diagnosis of gingivitis may be more likely to be examined for cancer. On the other hand, we also cannot exclude that gingivitis is only one consequence of a health-adverse lifestyle, meaning that risk factors accumulating in patients with gingivitis might lead to cancer in these patients.

In summary, we present data from a large UK primary care provider database showing that chronic gingivitis is associated with an increased incidence of cancer, which is most prominent in middle-aged men. Thus, along with previous data, our study highlights that the clinical management of patients with chronic gingivitis should include a careful and structured work-up of cancer in order to recognize malignancies at the earliest possible time point. Chronic gingivitis should be recognized as a systemic disease that can negatively affect the prognosis of patients far beyond local complications. Chronic gingivitis thus represents a complex and interdisciplinary challenge whose management requires structured cooperation between different medical disciplines such as dentists, internists, and oncologists to improve long-term outcomes in patients with chronic gingivitis. Given our finding of a significant association between gingivitis and prostate cancer, our findings suggest that the diagnosis of gingivitis typically collected in outpatient dental practices should encourage dentists to remind their male patients of the possibilities of systematic cancer screening programs and to document this.

## 5. Conclusions

Our data suggest that chronic gingivitis represents an important risk factor for the development of cancer. Therefore, in the context of patient dental care, awareness should be raised to refer gingivitis patients to existing screening programs, especially for prostate cancer. Moreover, the consistent treatment of gingivitis could potentially have a positive impact on the morbidity of certain cancers.

## Figures and Tables

**Table 1:** Baseline characteristics of study patients after 1:1 matching.

Variable	Patients with Chronic Gingivitis (n = 9891)	Patients without Chronic Gingivitis (n = 9891)	*p*-Value
*Sex*			
Men	4259 (43.1)	4259 (43.1)	1.000
Women	5632 (56.9)	5632 (56.9)	
*Age*			
Age in years (mean, standard deviation)	41.6 (17.6)	41.6 (17.6)	1.000
Age 18–40 years	5325 (53.8)	5325 (53.8)	1.000
Age 41–60 years	2857 (28.9)	2857 (28.9)	
Age >60 years	1709 (17.3)	1709 (17.3)	
*Charlson Comorbidity Index* (mean, standard deviation)	0.4 (0.8)	0.4 (0.8)	1.000

Data are absolute numbers and percentages unless otherwise specified.

**Table 2:** Association between chronic gingivitis and the incidence of cancer in patients followed for up to 10 years in general practices in the United Kingdom.

Population	Proportion in Patients with Chronic Gingivitis	Proportion in Patients without Chronic Gingivitis	Hazard Ratio (95% Confidence Interval)	*p*-Value
Overall	6.1	4.6	1.36 (1.15–1.62)	<0.001
Men	6.0	4.1	1.46 (1.13–1.89)	0.004
Women	6.2	5.0	1.29 (1.03–1.62)	0.029
Age 18–40 years	1.1	0.9	1.51 (0.85–2.67)	0.161
Age 41–60 years	8.4	4.4	1.74 (1.30–2.32)	<0.001
Age >60 years	18.2	16.6	1.19 (0.95–1.50)	0.136

## Data Availability

The datasets used and analyzed during the current study are available from the corresponding author on reasonable request.
